# Practical and Experimental Chemistry, Adapted to Arts and Manufactures

**Published:** 1838-10

**Authors:** 


### ART. XV.

*Practical and Experimental Chemistry; adapted to Arts and Manufactures.* By E. MITSCHERLICH, Professor of Chemistry, &c. *Translated from the first Portion of his Compendium, by* STEPHEN LOVE HAMMICK, M.D.—*London,* 1838. pp. 311.

THE Manual of Chemistry of the justly celebrated Berlin professor has been long known to the scientific world, and we need hardly say that it has added considerably to the reputation which he previously enjoyed. In a former Number we had occasion to notice the translation of this work into French. An English version is now before us; and we wish it were in our power to speak as favorably of it as of the excellent original; but, with every disposition to look with a lenient eye upon what is evidently a first attempt on the part of the translator, our duty compels us to say that Dr. Hammick has scarcely shown himself competent for the task. We have here about one half of the first volume; and, as the translator will doubtless proceed with the work, we trust he will receive in good part the few remarks which we feel compelled to offer.

To all acquainted with the original work, it must be obvious that, for a good translation of it, there is required an excellent knowledge of the German language and of the science of chemistry. In his preface, the translator makes an apology for harshness of style, which he ascribes to his having adhered to a literal translation. Now, however proper it may be to adhere closely to the original text, a translator is never justified in sacrificing the true sense of a passage to such an object. This Dr. Hammick has frequently done, so that in many places his style is not only harsh, but perfectly unintelligible. We shall not specify passages, because we are satisfied that all who read his volume will have no difficulty in finding them. We are surprised likewise to observe many grammatical errors: but, serious as these faults are, there are still greater on the subject of chemistry itself. He states in the preface that Reaumur's thermometer is "generally used in foreign schools." Our experience would lead us to say that the centigrade is more generally employed; and certainly, in all the standard modern works on chemistry, both in French and German, this is the thermometer used by the authors. Professor Mitscherlich, among others, employs the centigrade; but the translator has calculated nearly all the temperatures by Reaumur, in raising them to Fahrenheit's scale. Thus, phosphorus is described as melting at 160°, and the boiling point of ether is stated to be 112°; mistakes which we should have thought the slightest reflection upon the known chemical properties of these bodies would have prevented. The error extends, so far as we have seen, throughout the whole volume; and its occurrence is the more remarkable, since (at p. 42) Mitscherlich speaks of the boiling point of water as being at 100°, which Dr. Hammick translates 212°; but, in the same sentence, (p. 40, translation,) while Mitscherlich describes sulphur as boiling at 420°, (i. e. 578° F.) the translator makes the temperature 970°; an error of 400°. Probably Dr. Hammick has by this time discovered his mistake. We have no doubt that in the second volume he will guard against this; and, if he will receive from us a word of friendly advice, he will not adhere so literally to the original as to lose the author's meaning: he will be a little more careful in his grammar, and will submit the translation to the critical judgment of some friend, practically acquainted with chemistry, before he presents it to the public.

---

